# Singlet Oxygen-Mediated Oxidation during UVA Radiation Alters the Dynamic of Genomic DNA Replication

**DOI:** 10.1371/journal.pone.0140645

**Published:** 2015-10-20

**Authors:** Dany Graindorge, Sylvain Martineau, Christelle Machon, Philippe Arnoux, Jérôme Guitton, Stefania Francesconi, Céline Frochot, Evelyne Sage, Pierre-Marie Girard

**Affiliations:** 1 CNRS UMR 3348, Stress Génotoxiques et Cancer, Orsay, France; 2 Curie Institute, PSL Research University, Orsay, France; 3 Centre Hospitalier Lyon-Sud, Hospices Civils de Lyon, Laboratoire de biochimie-toxicologie, Pierre Bénite, France; 4 Laboratoire de chimie analytique, Université Lyon 1, ISPBL, Faculté de pharmacie, Lyon, France; 5 Université de Lorraine, Laboratoire Réactions et Génie des Procédés (LRGP), Nancy, France; 6 CNRS, UMR7274, Nancy, France; 7 Laboratoire de Toxicologie, Université Lyon 1, ISPBL, Faculté de pharmacie, Lyon, France; Cornell University, UNITED STATES

## Abstract

UVA radiation (320–400 nm) is a major environmental agent that can exert its deleterious action on living organisms through absorption of the UVA photons by endogenous or exogenous photosensitizers. This leads to the production of reactive oxygen species (ROS), such as singlet oxygen (^1^O_2_) and hydrogen peroxide (H_2_O_2_), which in turn can modify reversibly or irreversibly biomolecules, such as lipids, proteins and nucleic acids. We have previously reported that UVA-induced ROS strongly inhibit DNA replication in a dose-dependent manner, but independently of the cell cycle checkpoints activation. Here, we report that the production of ^1^O_2_ by UVA radiation leads to a transient inhibition of replication fork velocity, a transient decrease in the dNTP pool, a quickly reversible GSH-dependent oxidation of the RRM1 subunit of ribonucleotide reductase and sustained inhibition of origin firing. The time of recovery post irradiation for each of these events can last from few minutes (reduction of oxidized RRM1) to several hours (replication fork velocity and origin firing). The quenching of ^1^O_2_ by sodium azide prevents the delay of DNA replication, the decrease in the dNTP pool and the oxidation of RRM1, while inhibition of Chk1 does not prevent the inhibition of origin firing. Although the molecular mechanism remains elusive, our data demonstrate that the dynamic of replication is altered by UVA photosensitization of vitamins *via* the production of singlet oxygen.

## Introduction

The ultraviolet A (UVA) radiation (λ = 320–400 nm) is the main component of the UV radiation that reaches the surface of the Earth, and consequently our skin [1]. The primary cytotoxic effects of UVA radiation are due to the production of reactive oxygen species (ROS) by photosensitization. This process is initiated by the absorption of UVA photons by various endogenous (*e*.*g*. flavins, porphyrins, melanin) or exogenous (*e*.*g*. non steroidal anti-inflammatory drugs) photosensitizers [2–5]. There are two major pathways for photosensitization: Type I and Type II [6]. In the Type I reaction, the excited photosensitizer abstracts an electron/hydrogen atom from another component of the system. In the presence of molecular oxygen (^3^O_2_), the photosensitizer radical transfers the electron/hydrogen to ^3^O_2_, leading to the formation of the superoxide radical (O_2_^●-^). Superoxide can undergo spontaneous or enzymatic dismutation to generate hydrogen peroxide (H_2_O_2_), which can produce the highly toxic hydroxyl radical (HO^●^). In the Type II reaction, the excited photosensitizer transfers its energy to the ground state of ^3^O_2_ leading to the formation of singlet oxygen (^1^O_2_). UVA-induced ROS can cause oxidative damage to proteins, DNA and lipids [7–10], and the extent of these damages will depend on the concentration of the photosensitizer and/or its cellular localization.

DNA replication is required for faithful inheritance of the genome at each cell division. It starts by recognition of the replication origins by the ORC complex, which is composed of six proteins (Orc1 to Orc6) [11]. The binding of ORC complex to the origins allows the recruitment, during late mitosis and early G1, of the replication initiators Cdc6 (Cell division cycle 6) and Cdt1 (Chromatin licensing and DNA replication factor 1), which facilitates the binding of the core of the replicative DNA helicase Mcm2-7 in an inactive form (reviewed in [12–14]). These steps lead to the formation of a pre-replicative complex (pre-RC), commonly known as origin licensing, which is converted into a pre-initiation complex (pre-IC) by the activation of the CDKs (Cyclin-dependent kinases) and DDK (Dbf4-dependent kinase Cdc7) at the G1/S transition [12,13]. Activation of the CDKs and DDK allows the recruitment of additional factors including the GINS complex, Mcm10, RPA, DNA polymerase α, which initiate the origin firing (for reviews, see [15–18]). Recent works indicate that the time at which an origin fires is related to its ability to recruit replication initiation factors, such as Cdc45 and Sld3 (yeast ortholog of human Treslin/ticrr), which are limiting within the cells (see [19] and references therein). The current model is that firing factors are recruited to a subset of licensed origins leading to their activation, then are recycled on other licensed origins, which are in turn fired, and so on, throughout S-phase, until completion of DNA replication [19]. The loading of the replicative DNA polymerases Pol delta (Polδ) and Pol epsilon (Polε) in association with other replicative factors (*e*.*g*. Replication Factor C, PCNA) allows DNA synthesis [20]. Finally, the ATR/Chk1-mediated checkpoint pathway regulates initiation and progression of DNA synthesis in an unperturbed S-phase but also in the presence of DNA damage [21–23]. Thus, regulation of the S phase program occurs at several stages, affecting origin firing, fork velocity, and fork stability.

The replication forks elongation requires the incorporation of the deoxyribonucleotide triphosphates (dNTPs) by the replicative DNA polymerases. Thus, one limiting factor to nascent DNA strands elongation is the availability of each of the four dNTPs. The ribonucleotide reductase (RNR), or ribonucleotide diphosphate reductase (rNDP reductase), catalyses the first reaction committed to DNA synthesis, and is involved in the *de novo* synthesis of the four dNTPs [24,25]. The dNTPs are essential not only for genomic and mitochondrial DNA replication, but also for DNA repair. Therefore, the activity of RNR has to be finely regulated to maintain the steady-state level of the intracellular pool of dNTPs or to adjust it if necessary [25,26]. Moreover, while in yeast, the pool of dNTPs increases significantly in response to DNA damage, it remains almost unchanged in mammals [27].

The RNR is a heterotetramer composed of two large and two small subunits. In mammalian cells, there are a the large subunit RRM1, encoded by the gene *Rrm1* and two small subunits, RRM2 and RRM2B (also called p53R2), encoded by the genes *Rrm2* and *Rrm2B*, respectively [24]. In dividing cells, the main complex is formed by the association of RRM1 and RRM2 subunits, while in resting cells, the RRM1/p53R2 complex is predominant. Furthermore, this latter complex is thought to ensure mitochondrial DNA replication [28]. In mammals, RNR resides predominantly in the cytosol in the absence or presence of DNA damage [28–30], even though RNR recruitment at DNA damage sites requires the interaction of the RRM1 subunit with Tip60 histone acetyltransferase [31]. RNR activity is also regulated by its redox state. Indeed, in eukaryotes, the reduction of rNDPs by RNR involves the formation of a disulfide bond in the active site of the RRM1 subunit, which is subsequently reduced by a dithiol exchange reaction usnig the combined action of another pair of thiols located in the C-terminal tail of RRM1 and the thioredoxin/glutaredoxin systems [32].

We have previously shown that UVA-induced ROS led to inhibition of DNA synthesis by a mechanism that does not require a functional DNA damage checkpoint response [33]. To get insight into the DNA replication parameters affected by UVA-induced ROS, we used DNA molecular combing [34] to measure the fork velocity and origin density, high performance liquid chromatography coupled to mass spectrometry (HPLC-MS/MS) to determine the intracellular concentration of each of the four dNTPs [35] and Western blotting to study the relocalization or the post-translational modifications of some of the proteins involved in DNA replication. We found that generation of singlet oxygen upon UVA radiation can alter the dynamic of replication by affecting both the replication fork velocity and the origin firing.

## Materials and Methods

### Cell lines and silencing of gene expression (siRNA)

MRC5Vi is a SV40-transformed and immortalized cell line derived from the normal human lung fibroblasts MRC5 [36]. Eagle’s Minimum Essential Medium (MEM) with Earle’s salts containing phenol red and L-glutamine, L-glutamine (L-glu) 100X, penicillin 10000 UI/Streptomycin 10000 μg 100X (P/S), non-essential amino acid (NEAA) 100X, sodium pyruvate (100X), and fetal bovine serum (FBS) were from Eurobio (France). MEM without phenol red and L-glutamine (MEMi), MEM Vitamin Solution (100X) and MEM Amino Acids Solution (50X) were from Life Technologies. The cells were grown in 10% FBS Eagle’s MEM containing P/S 1X, L-glu 1X, NEAA 1X and sodium pyruvate 1X at 37°C, 95% humidity and 5% CO_2_. Transfection of human *TRX1* siRNA (*siTrx1*, 5’UCAGGAUGUUGCUUCAGAGUGUGAA3’), of human *GRX1* siRNA (*siGrx1*; 5’AAUUCCAGAAGCCCUUGUUUGAUGG3’), of human CHK1 siRNA (*siChk1*, 5’UCGCAGUGAAGAUUGUAGAUAUGAA3’) and of RNAi negative control low GC (*siCtr*) was performed in OptiMEM using INTERFERin (Polyplus-transfection, Ozyme, France). All siRNA were from Invitrogen Life Technology. The final concentration of siRNA was 10 nM and experiments were carried out 48h post transfection.

### Reagents

Aphidicolin was dissolved in DMSO. Buthionine sulfoximine (BSO), N-acetyl-L-cysteine (NAC), and sodium azide (NaN_3_) were dissolved in H_2_O. N-ethylmaleimide (NEM) was dissolved in 100% EtOH. All chemicals were purchased from Sigma-Aldrich (France).

### Cell irradiation

The cells were seeded in 40-mm, 60-mm or 100-mm dishes, rinsed twice with PBS or MEMi and irradiated in 1 mL, 2 mL or 5 mL, respectively, of PBS or MEMi using a Sellamed 4000 system that emitted principally in the spectral range from 340 to 440 nm (see www.sellamed.com for more information). Uncovered dishes were placed at a distance of 20 cm from the UVA source and the irradiance was 50 mW/cm^2^ (irradiation lasted less than 6 min). When indicated in the Figure legend, cells were irradiated in MEMi containing 10 mM NaN_3_. Following radiation, the medium was removed, the cells were rinsed twice with fresh complete medium and incubated at 37°C for the indicated period of times. For synchronisation in early S-phase, the cells were incubated for 16–18 h in the presence of 2.5 μg/μL of aphidicolin [37]. As indicated in the Figure legend, cells were either irradiated immediately (condition S0R) or at 4h (condition S4R) after release from aphidicolin.

### Luminescence experiments for the detection of singlet oxygen (^1^O_2_)

The absorption spectra were recorded either on a UVIKON XS Secomam (BioServ, France) or on a Perkin–Elmer Lambda EZ 210 (Courtaboeuf, France) UV-visible double beam spectrophotometer. Absorption was measured in standard quartz cuvettes with an optical path of 1 cm (104.002B-QS, Hellma Analytics, Germany). Fluorescence spectra were recorded on a Fluorolog FL3-222 spectrogfluorimeter (HORIBA Jobin Yvon, Longjumeau, France) equipped with a 450 W Xenon lamp, a thermostated cell compartment (25°C), a UV-visible photomultiplier R928 (HAMAMATSU Japon) and an InGaAs infrared detector (DSS-16A020L Electro-Optical System Inc, Phoenixville, PA, USA). The excitation beam was diffracted by a double ruled grating SPEX monochromator (1200 grooves/mm blazed at 330 nm). The emission beam was diffracted by a double ruled grating SPEX monochromator (1200 grooves/mm blazed at 500 nm). Singlet oxygen emission was detected through a double ruled grating SPEX monochromator (600 grooves/mm blazed at 1 μm) and a long-wave pass (780 nm). All spectra were measured in four-face quartz cuvettes. All the emission spectra (fluorescence and singlet oxygen luminescence) have been normalized with the same absorbance, with the lamp and photomultiplier correction.

### BrdU incorporation and cell cycle analysis

The protocol used has been extensively described [33]. Briefly, the cells were pulse-labeled with 10 μM BrdU for 30 min either before or after UVA radiation, as described in the Figure legend, fixed in cold 70% EtOH, and then treated with FITC-conjugated anti-BrdU antibody to label S-phase cells, and with propidium iodide (PI) to evaluate DNA content. All samples were analysed by a FACSCalibur flow cytometer (Becton Dickinson, BD, France).

### Depletion of GSH

Depletion of intracellular glutathione (GSH) was performed by adding BSO to the cell culture, as previously described [38].

### Molecular combing and statistical analysis

Molecular combing was performed as described [39]. Briefly, MRC5Vi cells, asynchronous or synchronized in S-phase, were exposed to UVA radiation, and the IdU and CldU labeling (30 min pulse labeling at 100 μM each) was performed either immediately or at various time points post radiation. At the end of the CldU pulse, trypsinized cells were embedded in low-melting agarose plugs. After digestion with ß-agarase (NewEngland Biolabs), DNA was combed on silanized surfaces (provided by Genomic Vision, France or by the Montpellier DNA combing facility, France). The nucleoside analogs were revealed by incubating coverslips for 1 hour with mouse anti-BrdU FITC antibody (1/5; Becton Dickinson) and rat anti-BrdU antibody (1/25; AbD Serotec). After several washing steps with PBS, coverslips were incubated with secondary antibodies: Alexa-Fluor-488-conjugated goat anti-mouse and Alexa-Fluor-594-conjugated goat anti-rat (1/25 each; Molecular Probes, Life Technologies). After several washes, coverslips were sequentially incubated with three antibodies to further detect single-stranded DNA (ssDNA): mouse anti-ssDNA (1/100; Millipore), rabbit anti-mouse Alexa-Fluor-350 and goat anti-rabbit Alexa-Fluor-350 (1/50 each; Molecular Probes). All antibodies were diluted in BlockAid solution (Invitrogen, Life Technologies). Finally, coverslips were mounted using mounting media Prolong Gold Antifade Reagent (Invitrogen). A tiled scan of the coverslips was performed on a Leica DM RXA fluorescent microscope (Leica Microsystemes SAS) coupled with a CoolSNAP HQ camera (Photometrics) and the images captured with Metamorph software (Molecular Devices). Tiled images were reconstructed using ImageJ (open source from the National Cancer Institute, NIH) with the Stitching Plugin (2d-5d) [40], and the signals were measured. To determine the density of replication origins, we counted the number of symmetrical green-red signals for a total DNA length of 100–300 Mb. Then, we estimated, by BrdU labeling and flow cytometry analysis, the percent of DNA corresponding to cells in S-phase. Distributions of replication fork velocities were compared by means of the Mann-Whitney test.

### Measurement of intracellular concentration of dNTPs

Quantification of dATP, dGTP, dCTP and dTTP was carried out using liquid chromatography coupled with a tandem mass spectrometer (LC-MS/MS) and based on a previously published assay. Briefly, an on-line solid phase extraction on an Oasis WAX column (3.9 mm x 20 mm; 30 μm—Waters) was performed, followed by the separation of the compounds thanks to a Hypercarb analytical column ((2.1 mm x 100 mm; 5 μm—ThermoScientific). A gradient was programmed using varying quantities of NH_4_OH 0.25% pH10, water and acetonitrile. An electrospray ionisation (ESI) source was used, and a positive mode selected for the detection of the compounds. Quantification was performed by adding standard solutions of labeled nucleotides to cellular samples (2’-deoxyadenosine-^13^C_10_,^15^N_5_ 5’-triphosphate, 2’-deoxycytidine-^13^C_9_,^15^N_3_ 5’-triphosphate, 2’-deoxyguanosine-^13^C_10_,^15^N_5_ 5’-triphosphate and thymidine-^13^C_10_,^15^N_2_ 5’-triphosphate). Thus, concentrations of endogenous nucleotides were calculated using calibration curves of the corresponding labeled nucleotides. Adenosine-^13^C_10_ 5’-triphosphate (ATP_13C_) and cytidine-^13^C_9_ 5’-triphosphate (CTP_13C_) were used as internal standards. The assay was fully validated in term of accuracy, precision, selectivity and stability [35].

### Recovery of soluble and chromatin-bound protein extracts

Untreated and treated cells were washed with cold PBS and lysed on ice for 5 min in lysis buffer [10 mM HEPES, pH 7.5, 100 mM NaCl, 300 mM sucrose, 3 mM MgCl_2_, 1 mM EGTA, 50 mM NaF, 20 mM ß-glycerophosphate, 0.3% Triton X-100, 0.1 mM sodium orthovanadate and complete mini EDTA-free protease inhibitors (Roche Diagnosis)]. When indicated in the Figure legend, 10 mM NEM were added to the lysis buffer to react with thiol groups to prevent their oxidation during protein extraction and protein analysis by SDS-PAGE [38]. At this step, the lysis buffer contained the total amount of soluble proteins, the amount being quantified by a Bradford assay. The proteins were precipitated by adding 5 volumes of 100% cold acetone. After removal of acetone and air drying, the pellets were dissolved in 1.5 X SDS loading buffer (150 mM Tris-HCl, pH 6.8, 3% SDS, 0.15% bromophenol blue, 15% glycerol, +/- 150 mM ß-mercaptoethanol) and denatured for 5 min at 95°C. The chromatin-bound fractions were recovered by scraping off the nuclear matrix from the plates in lysis buffer. After centrifugation at 4°C, chromatin-bound fractions were resuspended in 1.5 X SDS loading buffer containing ß-mercaptoethanol, denatured for 15 min at 95°C and centrifuged for 30 min at room temperature to pellet any solids.

### Western blot analysis

The protein extracts were separated by SDS-PAGE and transferred onto PROTRAN^®^ nitrocellulose membranes (Whatman) using a Trans-Blot Semi-Dry apparatus (Bio-Rad Laboratories). The membranes were probed with the following primary antibodies from Santa Cruz Biotechnology (SCBT), Cell Signaling Technology (CST), Calbiochem, Abnova, Abcam, Bethyl Laboratories (BL), Novus Biologicals (NB), or Sigma-Aldrich (SA), and raised against Lamin A/C (E-1, SCBT), GAPDH (A-3, SCBT), Actin (AC-15, SA), Chk1 (G-4, SCBT), phospho-Chk1 Ser345 (133D3, SCBT), ATR (N-19, SCBT), PCNA (PC10, SCBT), RPA32 (ab2626, Abcam), Cdc7 (DCS-341, NB), Cdc6 (DCS-180, NB), Dbf4 (NBP1-68173, NB), Mcm2 (D7G11, CST), phospho-Mcm2 S40/S41 (A300-788A, BL), Mcm10 (NB100-253, NB), Cdc45 (3673, CST), Orc2 (H-300, SCBT; AbVantage™ Pack, BL), p53R2 (ab8105, Abcam), RRM2 (165–174, Calbiochem), RRM1 (D12F12 or 3388, SCBT), Grx1 (3C11, Abnova) and Trx1 (8A1, SCBT). Primary antibodies were diluted in Tris buffered saline (TBS) containing 5% BSA and 0.05% Tween 20. The membranes were then probed with the appropriate peroxidase-conjugated secondary antibody diluted in TBS containing 5% non fat dry milk and 0.05% Tween 20. The signal detection was performed using the ECL Western blotting Detection Reagents (Amersham Biosciences) or the WesternBright ECL-spray (ECL^+^, Advansta, France) combined with Hyperfilm autoradiography films that were developped on a Curix 60 film processor (AGFA). The films were further scanned on a Pro48 scanner (PFU, Japan) controlled by the *SilverFast Ai* scan software (LaserSoft Imaging AG, Germany).

## Results

### Singlet oxygen is produced in the UVA-irradiated medium (MEMi) *via* photosensitization of vitamins

**Fig 1:**
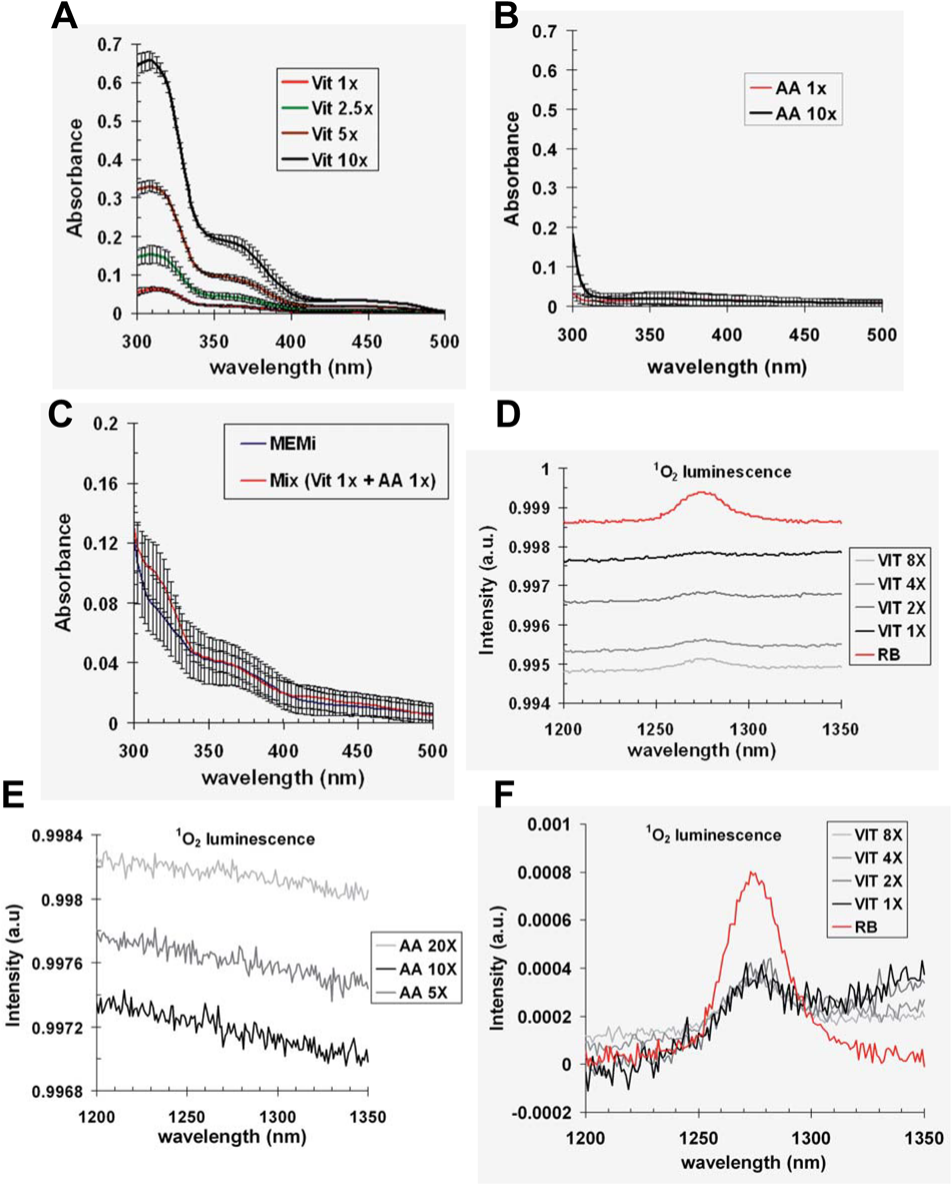
Singlet oxygen is generated by photosensitization of vitamins. Absorption spectra between 300 and 500 nm of various dilutions in H_2_O of (**A**) vitamins (Vit) and (**B**) amino acids (AA). (**C**) Comparison between the absorption spectrum of a mixture of vitamins 1x and amino acids 1x diluted in H_2_O (Mix Vit1x + AA1x) and the absorption spectrum of MEMi. The stock solutions of vitamins and amino acids were 100x and 50x, respectively. Each spectrum is the average ± SD of 3 to 4 independent spectra. Solutions of AA (**D**) and Vit (**E**) were diluted in D_2_O and excited at 370 ± 7 nm. The ^1^O_2_ luminescence of excited solutions was recorded as described in Material and Methods. (**F**) The ^1^O_2_ luminescence of each dilution of Vit was normalized with the appropriate absorbance at 370 nm, and is compared to the ^1^O_2_ luminescence of a solution of Rose Bengal (RB).

We previously reported that exposure of cells to UVA radiation in the culture medium (MEMi) led to the slowdown of genomic DNA replication by a mechanism that relies on the generation of ROS [33]. This medium is a mixture of amino acids, vitamins, inorganic salts and glucose, and we showed that absorption of UVA photons by vitamins but not amino acids mostly contribute to this slowdown [33]. N-acetyl-L-cysteine (NAC), a scavenger of hydroxyl radical (HO^•^) and hydrogen peroxide (H_2_O_2_) [41], partially prevented the delay [33] showing that UVA-dependent generation of these reactive oxygen species (ROS) contributes to some extent to this effect. However, photosensitization of vitamins by UVA also generates singlet oxygen (^1^O_2_) [2,42]. To demonstrate that, in our experimental conditions, ^1^O_2_ contributes to the overall delay of replication, we decided to monitor its production by measuring its radiative relaxation to its ground state at 1270 nm [4,42]. At first, we recorded the absorption spectra between 300 and 500 nm of 10x to 1x concentrated solutions of vitamins and amino acids. The vitamin solutions (Fig 1A), but not the amino acid solutions (Fig 1B), efficiently absorb in the UVA range with a shoulder around 360–370 nm (Fig 1A). In fact, the absorption spectrum of a mixture of amino acid 1x and vitamins 1x (concentrations found in MEMi) closely resembles the absorption spectrum of MEMi (Fig 1C). Based on these observations, ^1^O_2_ luminescence was recorded in solutions of vitamins and amino acids prepared in D_2_O (to increase the lifetime of ^1^O_2_ [43,44]), and exposed to a spectral bandwidth of 370 ± 7 nm (see Material and Methods). As a control, we used a solution of Rose Bengal (RB), well-known to generate ^1^O_2_ *via* photosensitization [45]. A clear signal centred on 1270 nm was detected using the solutions of vitamins and the solution of RB (Fig 1D), while no signal was detected with amino acid solutions (Fig 1E). However, no ^1^O_2_ luminescence was recorded in MEMi (data not shown), most likely due to the fact that the half-lifetime of ^1^O_2_ in H_2_O is extremely short [43,44]. Taking into account the absorbance at 370 nm of each dilution of vitamins, we showed that the signal of ^1^O_2_ luminescence is directly proportional to the concentration of vitamins (Fig 1F). From these data, the singlet oxygen quantum yield of vitamins in D_2_O was 22 ± 3%, compared to 76% for RB [46]. These results demonstrate that ^1^O_2_ is produced by photosensitization of vitamins, but not of amino acids, at an excitation wavelength of 370 ± 7 nm, which is only a narrow subset of the total UVA spectrum emitted by our lamp.

### Photosensitization by UVA induces an immediate and prolonged S-phase delay that is partially suppressed by sodium azide

**Fig 2:**
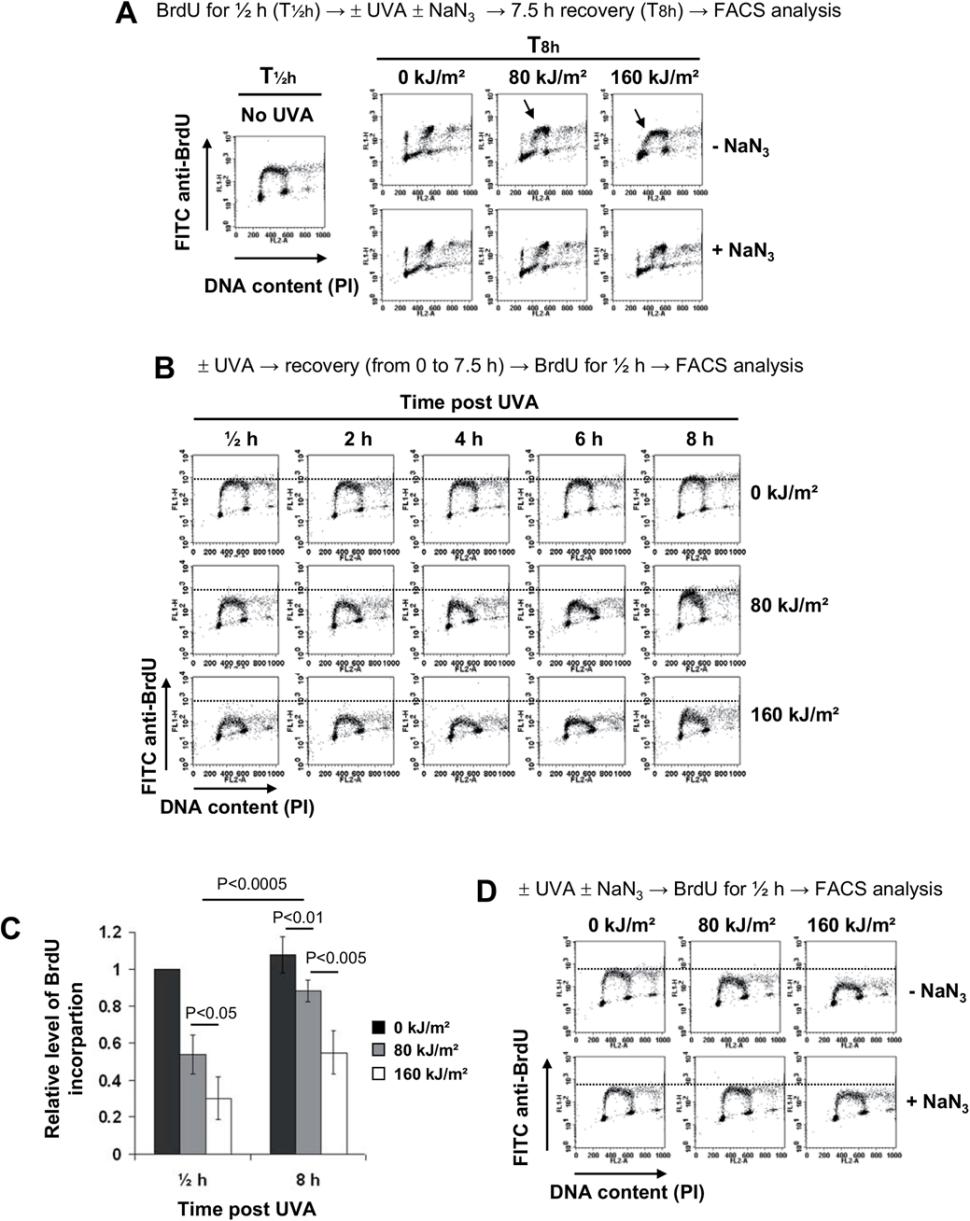
UVA radiation induces S-phase slowdown that is prevented by NaN_3_, a quencher of singlet oxygen. (**A**) Transformed human fibroblasts (MRC5Vi) were pulse-labeled with 10 μM BrdU for 30 min (T½h) and exposed to UVA radiation in MEMi containing or not 10 mM NaN_3_ or 10 mM NAC. Thereafter, the cells were incubated at 37°C for 7.5 h and fixed in cold 70% EtOH (T8h). (**B**) The cells were exposed to UVA radiation in MEMi, incubated for different periods of time at 37°C, pulse-labeled with BrdU for 30 min, and fixed in cold 70% EtOH. (**C**) Histograms of the relative level of BrdU incorporation (FITC anti-BrdU axis) at T½h and T8h in non irradiated and irradiated cells displayed in panel B. The values represent the mean +/- SD of 3 to 5 independent experiments. Statistical significances were determined by Student *t*-test. (**D**) The cells were exposed to UVA radiation in MEMi containing or not 10 mM NaN_3_, immediately pulse-labeled with BrdU for 30 min, and fixed in cold 70% EtOH. The cells were analysed by bi-variable flow cytometry for BrdU incorporation (FITC anti-BrdU) and DNA content (propidium iodide, PI). The arrows highlight the delay of DNA replication (panels in A) and the dashed lines position the maximum level of BrdU incorporation in untreated cells (panels in B and D). It is to note that this level is lower in UVA-irradiated cells thus highlighting a defect of BrdU incorporation.

To evaluate the impact of UVA-induced ^1^O_2_ on the slowing down of DNA replication, we used sodium azide (NaN_3_), an effective quencher of singlet oxygen (^1^O_2_) [2,45,47,48]. Asynchronous MRC5Vi cells were pulse-labeled with BrdU, irradiated in MEMi containing NaN_3_ or not, and the progression of cells in S-phase was analysed 8 h post treatment. We found that irradiation of cells in MEMi led to a dose-dependent inhibition of DNA replication that was quite strongly prevented by NaN_3_ (Fig 2A). These results were confirmed using cells synchronised in S-phase (shown in S1 Fig).

As the ROS level increases during UVA radiation and returns to its basal level 1h later ([49]; shown in S2 Fig), we attempted to see if the slowdown of DNA replication was an early response to the radiation. MRC5Vi cells were left untreated or exposed to UVA radiation in MEMi, and pulse-labeled for ½h with BrdU at various time points (from 0 to 7h½) post radiation. We found a dose-dependent decrease of the level of BrdU incorporation immediately after UVA radiation (Fig 2B), a decrease that was quite efficiently suppressed by NaN_3_ (Fig 2D). BrdU incorporation dropped by 46% and 70% immediately after a dose of 80 and 160 kJ/m^2^ of UVA radiation, respectively, and by 12% and 46% eight hours after these doses (Fig 2B and 2C). Therefore, both the extent of the decrease and the time of recovery depend on the UVA dose. Furthermore, BrdU labeling performed 8 h after an irradiation at 80 or 160 kJ/m^2^ of UVA revealed two populations of cells in S-phase (Fig 2B): an early-to-mid S-phase population with a BrdU incorporation comparable to that of untreated cells (the beginning of a horseshoe-like shape) and a mid-to-late S-phase population with a diffuse BrdU incorporation reflecting altered DNA replication. The early-to-mid S-phase population corresponds to cells that were in G1 at the time of irradiation and the mid-to-late S-phase population corresponds to cells that were in S-phase at the time of irradiation. Together, these results demonstrate that DNA replication is immediately impaired after photosensitization by UVA radiation and remains altered several hours later.

### Photosensitization by UVA transiently inhibits the progression of the replication forks

**Fig 3:**
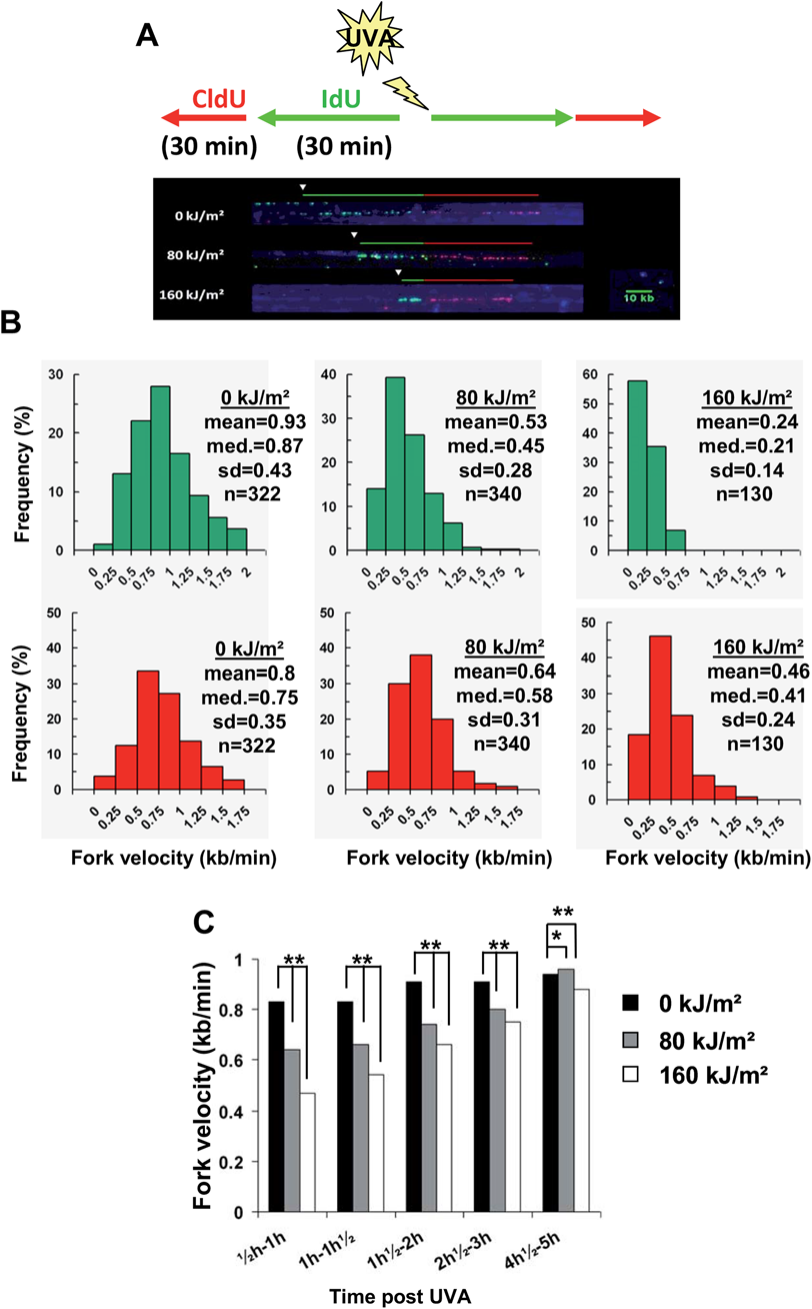
UVA-induced ROS impinge on the replication forks velocity. (**A**) Scheme of the experimental protocol. MRC5Vi cells were exposed or not to UVA and then pulse-labeled for 30 min of IdU (green signal) followed by 30 min of CldU (red signal). DNA was extracted immediately after the 2^nd^ pulse. Single stranded DNA (ssDNA) and incorporation of the thymidine analogs were detected as described in the Material and Methods. The picture is an example of the tracks length of IdU (green track), CldU (red track) and ssDNA (blue track). (**B**) Asynchronous MRC5Vi cells were untreated or exposed to 80 and 160 kJ/m^2^ of UVA radiation. IdU and CldU labeling was performed sequentially immediately after radiation. sd: standard deviation; med.: mediane; n: number of replication forks analysed. (**C**) The experiments were conducted as described in (B) with the exception that IdU (green) and CldU (red) labelings were performed at various time points (*i*.*e*. 0h, ½h, 1h, 2h and 4h) after UVA radiation. To determine the impact of UVA radiation on fork velocity, only the length of CldU tracks was scored. The values correspond to the mean of the forks velocity and are representatives of two experiments. A total of 500 to 3000 forks were analysed for these experiments. **P<0.001; *P<0.05 (two-tailed test).

To get insight into the molecular mechanism causing UVA-induced slowdown of replication, we used DNA molecular combing. Asynchronous populations of MRC5Vi cells in exponentially growing phase were left untreated or exposed to 80 and 160 kJ/m^2^ of UVA, and immediately pulse-labeled sequentially with IdU and CldU (30 min each). The combed DNA molecules were uniformly stained blue using anti-DNA antibody and the thymidine analogs were revealed by green and red fluorescence (see Material and Methods) (Fig 3A). This allowed us to calculate the speed at which the forks move by measuring the size of the labeled tracks. We observed a dose-dependent reduction in the length of each track, thus reflecting a slowdown of the fork velocity by UVA radiation (Fig 3B). We calculated that the mean fork velocity in unperturbed MRC5Vi cells was about 0.9 kb/min, while during the first 30 min after irradiation (IdU tracks), it was reduced by 43 and 74% in response to 80 and 160 kJ/m^2^ of UVA, respectively (Fig 3B). Analysis of the CldU tracks (time slots from 30 to 60 min after radiation) revealed that the mean fork velocity was reduced by 20 and 42% in response to 80 and 160 kJ/m^2^ of UVA, respectively.

To better assess this observation, we performed a time course analysis of the replication fork velocity in untreated and UVA treated cells. To do so, the IdU and CldU labelings (30 min each) were performed at various time points after UVA radiation, and we scored the CldU tracks to determine fork velocity. Consistently with the experiments described above, a recovery of the forks velocity over time was observed in UVA-treated cells (Fig 3C). We also performed these experiments in cells synchronized by aphidicolin and irradiated 4h after the release from aphidicolin block. We obtained similar results (shown in S3 Fig). Furthermore, irradiation of cells in PBS (data not shown) or in MEMi in the presence of NaN_3_ (shown in S3 Fig) did not trigger a reduction of the forks velocity. Collectively, these data suggest that the impact of UVA-induced ROS on the replication forks progression is transient.

### The dNTP pool is moderately and transiently affected by photosensitization

**Fig 4:**
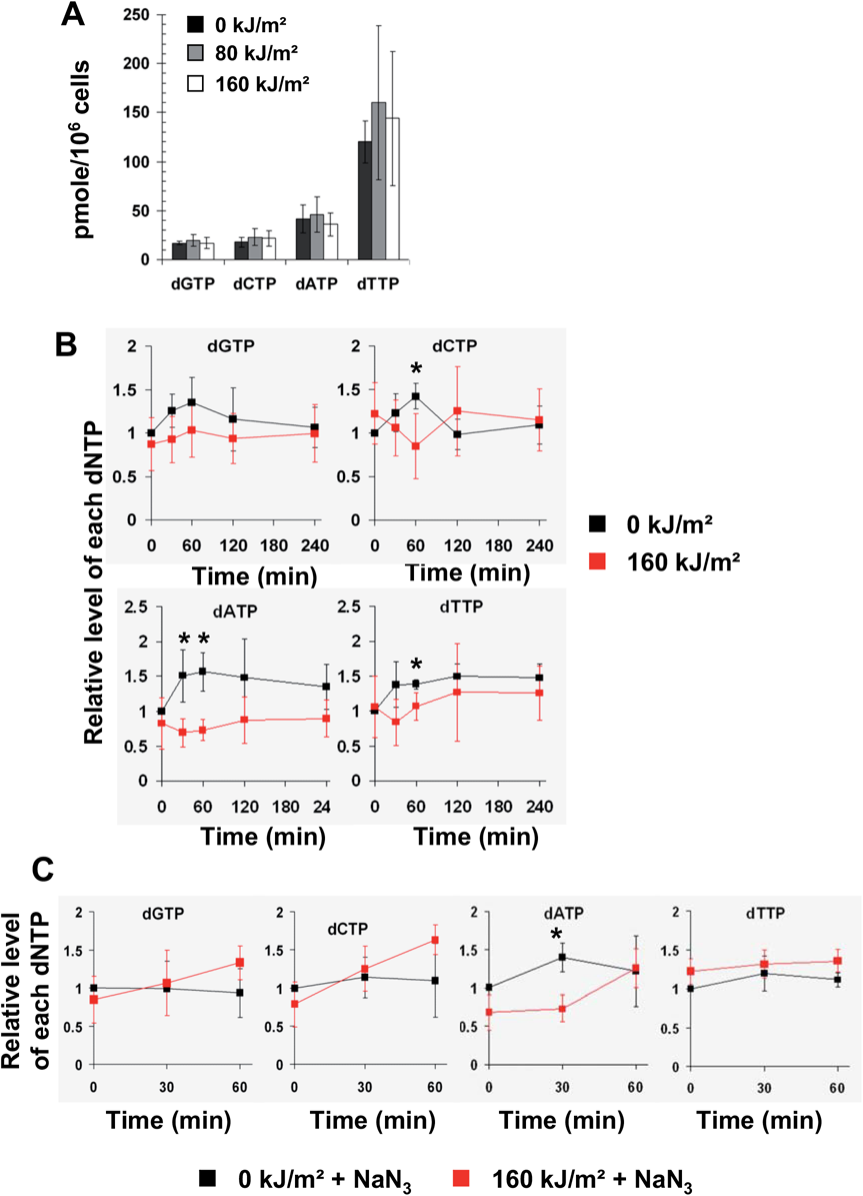
The transient drop of dNTP pool after photosensitization by UVA is prevented by NaN_3_. MRC5Vi cells in mid S-phase (condition S4R, see Material and Methods) were untreated or exposed to 80 and 160 kJ/m^2^ of UVA radiation in MEMi. The intracellular pool of dNTPs was measured at various time points post UVA radiation. (A) Quantification of dGTP, dCTP, dATP and dTTP in non irradiated cells and immediately after UVA radiation. The values represent the mean +/- sd of 5 independent experiments. (B) The relative level of each dNTP was measured at the indicated time points post UVA. The values represent the mean +/- sd of 5 independent experiments and were normalized to the value at T0–0 kJ/m^2^. (C) The cells were exposed to 160 kJ/m^2^ of UVA radiation in MEMi containing 10 mM NaN_3_. The relative level of each dNTP was measured at the indicated time points. The values represent the mean +/- sd of 3 independent experiments. *P<0.05 (*t*-test)

Since it has been shown that a reduction in deoxyribonucleotides availability leads to a reduction of the rate of replication fork progression [50,51], we wondered if UVA radiation induces a decrease of the dNTP pool in S-phase cells. MRC5Vi cells were synchronized by aphidicolin, treated or not by UVA radiation in mid S-phase (condition S4R, see Material and Methods), and collected at various time points after radiation in order to analyse the level of intracellular dNTPs. In untreated cells, the basal level of dGTP and dCTP was ≈ 20 pmoles per 10^6^ cells, of dATP ≈ 50 pmoles per 10^6^ cells and of dTTP ≈ 140 pmoles per 10^6^ cells, and these levels were unchanged immediately after UVA radiation (Fig 4A). These values are in good agreement with those obtained in cycling normal human fibroblasts [52]. In the first hour following 160 kJ/m^2^ of UVA, we observed a slight and transient reduction in the levels of dCTP, dATP and dTTP which was more pronounced with dATP, while there was no apparent decrease for dGTP (Fig 4B). Notably, these observations contrasted with the 1.5 fold increase in the level of each dNTP observed in non irradiated cells (Fig 4B). It should be noted that differences in the level of each dNTP between non irradiated cells and cells exposed to 80 kJ/m^2^ of UVA were not significant (shown in S4 Fig). To evaluate the role of UVA-induced ^1^O_2_ in the down regulation of dCTP, dTTP and dATP, the cells were exposed to 160 kJ/m^2^ of UVA in the presence of NaN_3_. We found that NaN_3_ completely prevented (for dCTP and dTTP) and strongly attenuated (for dATP) the initial decrease of these dNTPs in UVA irradiated cells (Fig 4C). Collectively, these results suggest that UVA-induced ROS moderately and transiently affect the expression level of the dNTP pool but at UVA doses > 80 kJ/m^2^.

### The RRM1 subunit of the ribonucleotide reductase is transiently oxidized by UVA-induced ROS

**Fig 5:**
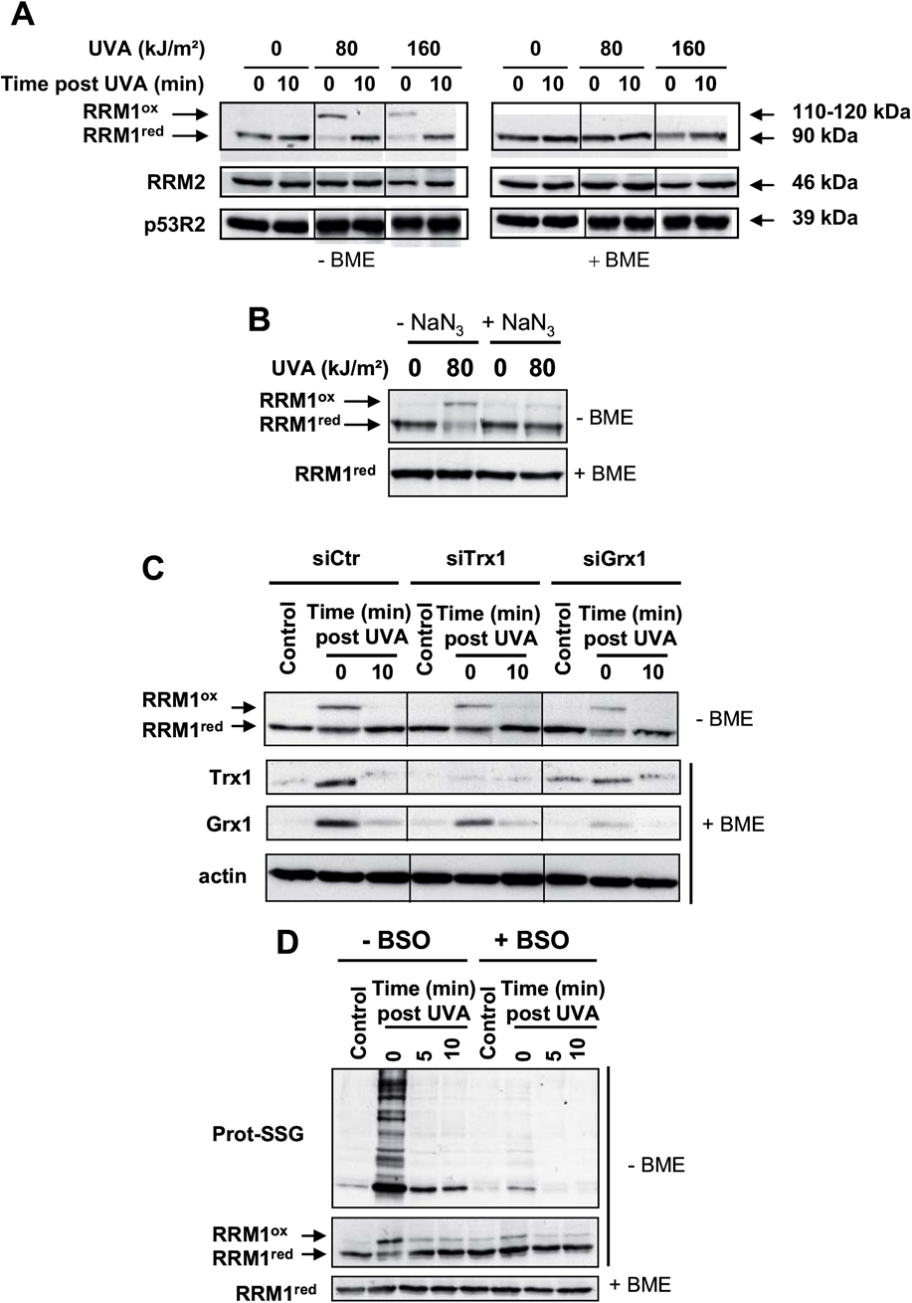
Reversible oxidation of the RRM1 subunit of RNR in response to UVA-induced ROS. MRC5Vi cells were untreated or exposed to UVA radiation in the absence (**A**) or presence (**B**) of 10 mM NaN_3_. (**C**) MRC5Vi cells were transiently transfected with siCtr, siTrx1, or siGrx1 and 48 h post transfection exposed or not to 80 kJ/m^2^ of UVA. (**D**) Cells depleted (+ BSO) or not (- BSO) in intracellular GSH were exposed to 80 kJ/m^2^ of UVA and lysed either immediately or 5 and 10 min post UVA radiation. The expressions of RRM1, RRM2, p53R2, Trx1, Grx1, and proteins S-glutathionylated (prot-SSG) were detected by Western blotting in non reducing (- ß-mercaptoethanol, - BME) or reducing (+ ß-mercaptoethanol, +BME) conditions. Actin was used as a loading control. RRM1^ox^ and RRM1^red^ stand for the oxidized and reduced form of the RRM1 subunit of the RNR, respectively. Each blot is representative of 2 independent experiments. The vertical lines in panels A and C denote non-adjacent bands from the same blot. Numbers in brackets (panel A) indicate the apparent molecular weight of each subunit.

As we observed a transient and moderate reduction of the intracellular reservoir of dNTP in the first hour after UVA radiation, we wondered if this effect reflected a transient inhibition of the ribonucleotide reductase (RNR) activity and if the enzyme was the target of UVA-induced ROS. By looking at the different subunits of the RNR (RRM1, RRM2 and p53R2) by Western blotting, we observed a change in the electrophoretic mobility of the RRM1 subunit towards higher molecular weights in UVA-treated samples run in non-reducing conditions. No apparent changes were detected for the RRM2 and p53R2 subunits (Fig 5A). This oxidized form of RRM1 was reduced *in vitro* by ß-mercaptoethanol or in cells in a few minutes after UVA radiation (Fig 5A). Furthermore, oxidation of RRM1 (RRM1^ox^) was triggered by UVA-induced ^1^O_2_ since the presence of NaN_3_ in MEMi during radiation largely reduced the formation of RRM1^ox^ (Fig 5B). We estimated the increase of RRM1 apparent molecular weight by 20 to 30 kDa. RRM1^ox^ may result from the formation of intermolecular disulfide bridges between the RRM1 subunit and an unknown protein, since bridges are efficiently reduced by intracellular reducing systems. Because RNR activity is regulated by the thioredoxin/glutaredoxin systems [53,54], we checked if the main cytosolic thioredoxin (Trx1, UniProtKB/Swiss-Prot P10599, apparent molecular weight of 12 kDa) or the main cytosolic glutaredoxin (Grx1, UniProtKB/Swiss-Prot P35754, apparent molecular weight of 12 kDa) were involved in the formation of RRM1^ox^ and/or in the reduction of RRM1^ox^. The expression of Trx1 or Grx1was down-regulated in MRC5Vi cells by transient transfection of *siTrx1* or *siGrx1*, respectively. We found that Trx1 or Grx1 are neither involved in the formation of RRM1^ox^ nor in its reduction since RRM1 was oxidized and reduced in Trx1 or Grx1 deficient cells, similarly to proficient cells (Fig 5C).

As we have recently shown that photosensitization by UVA radiation led to both GSH-independent and GSH-dependent formation of disulfide bonds in mammalian cells [38], we tested the role of GSH in RRM1 oxidation. To down-regulate the intracellular content of GSH, we used DL-buthionine-[*S*,*R*]-sulfoximine (BSO), an inhibitor of GSH biosynthesis [55,56], as previously reported [38]. To assess the efficient down-regulation of GSH, we checked by Western blotting the presence or not of protein S-glutathionylation, a process that results in the formation of mixed disulfides between proteins and GSH (prot-SSG) [57]. We found that the formation of both RRM1^ox^ and prot-SSG adducts by UVA photosensitization was strongly reduced in GSH-deficient cells compared to GSH-proficient cells (Fig 5D). This result suggests that intracellular GSH plays a role in the formation of RRM1^ox^ in response to UVA-induced ROS.

### Inhibition of the origin firing after photosensitization by UVA

**Fig 6:**
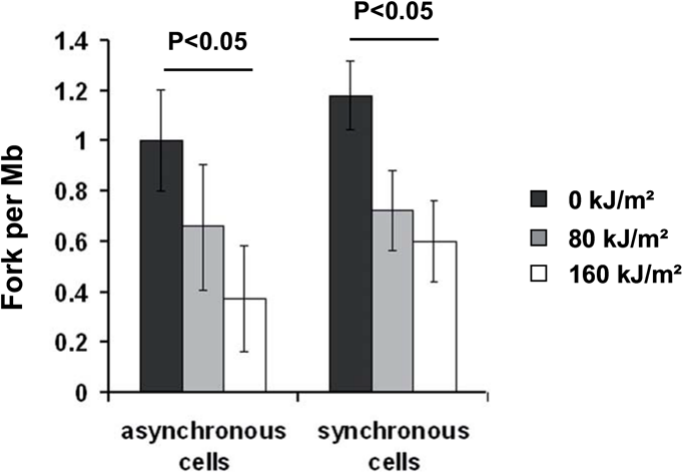
The firing of origins is reduced in response to UVA-induced ROS. Asynchronous or S-phase synchronised (condition S4R, see Material and Methods) cells were exposed to 80 and 160 kJ/m^2^ of UVA in MEMi. IdU and CldU were then sequentially added to the medium 4 h (asynchronous cells) or 2 h (S-phase cells) after UVA radiation. The forks density is defined as the ratio between the number of CldU-IdU-CldU signals (Red-Green-Red signals) and the length of replicated DNA. The percentage of cells in S-phase at the labeling time was determined by flow cytometry and was found to be 47% for the asynchronous population and 80% for S-phase synchronised cells. The data represent the mean ± sd of 4 independent experiments for A and 3 independent experiments for B (excepted for the dose of 80 kJ/m^2^ of UVA for which only 2 data were available).

Although we have shown that UVA-induced ROS led to a transient reduction of the forks velocity, this cannot fully explain the prolonged inhibition of DNA replication (see Fig 2). Therefore, we wondered if UVA-induced ROS also impinge on the firing of replication origins. The replication forks density was determined after UVA radiation in asynchronous cells and in cells synchronized in S-phase (condition S4R). We found that the mean fork density is 1.0 ± 0.2 and 1.18 ± 0.14 fork per Mb in non-irradiated asynchronous and synchronous cells, respectively (Fig 6). In asynchronous cells, 4 h after a dose of 80 and 160 kJ/m^2^ of UVA, the mean fork density was 0.65 ± 0.25 fork per Mb (*i*.*e*. 35% of inhibition) and 0.37 ± 0.21 fork per Mb (*i*.*e*. 63% of inhibition), respectively (Fig 6). Similar results were obtained in cells synchronized in S-phase. Indeed, 2 h after UVA radiation, the mean fork density was inhibited by 38% at 80 kJ/m^2^ of UVA and by 50% at 160 kJ/m^2^ of UVA (Fig 6). Such inhibitions were not observed if the cells were exposed in PBS to the same doses of UVA (data not shown). Collectively, these results suggest that UVA-induced ROS also inhibit origin firing.

It has been reported that activation of the ATR/Chk1 pathway inhibits origin firing [22,58], and we have previously shown that UVA-induced ROS activate the ATR/Chk1 pathway, although it is not required to trigger S-phase delay [33]. To demonstrate further the absence of Chk1 involvement in the inhibition of replication after UVA radiation, we determined the fork density in Chk1 proficient and deficient cells. In agreement with previously published data [21,59], the origin density was higher in non-irradiated Chk1 deficient cells (1.4 forks per Mb) compared to normal cells (0.80 fork per Mb) (Table 1). Inversely, the fork velocity was higher in Chk1 proficient than Chk1 deficient cells. However, we found a similar reduction of the origin density in Chk1 deficient and proficient cells (57% vs 55%, respectively) 4 h after radiation while the fork velocity was not reduced (Table 1). As previously reported [33], cell cycle analysis by flow cytometry of BrdU-labeled cells deficient for Chk1 showed a slowdown of DNA replication upon irradiation (shown in S5 Fig). In conclusion, these experiments demonstrate that the sustained inhibition of origin firing after UVA radiation does not rely on Chk1.

**Table 1:** Inactivation of origins firing in response to UVA-induced ROS in Chk1-deficient MRC5Vi cells.

siRNA	Dose of UVA (kJ/m^2^)	Number of double staining (red-green-red signals) scored	Total DNA length analysed (Mb)	Fork density (number of forks/Mb)	Percent of inhibition	Fork velocity (kb/min)
*siCtr*	0	80	180	0.80		1.34
*siCtr*	160	24	120	0.36	55	1.22
*siChk1*	0	85	110	1.40		0.91
*siChk1*	160	40	121	0.60	57	0.85

Asynchronous MRC5Vi cells were transfected with siCtr or siChk1 and exposed to 160 kJ/m^2^ of UVA in MEMi 48h post transfection. IdU and CldU were then sequentially added to the medium 4 h post UVA. The forks density is defined as the ratio between the number of CldU-IdU-CldU signals (red-green-red signals) and the length of replicated DNA. The percentage of cells in S-phase at the labeling time was determined by flow cytometry and was found to be 47%. The forks velocity was recorded only using the CldU tracks length. The data correspond to one experiment.

### The chromatin-bound fraction of Cdc6, Cdc45, Mcm2, Mcm10, Orc2, PCNA and RPA32 is not modified after photosensitization by UVA

**Fig 7:**
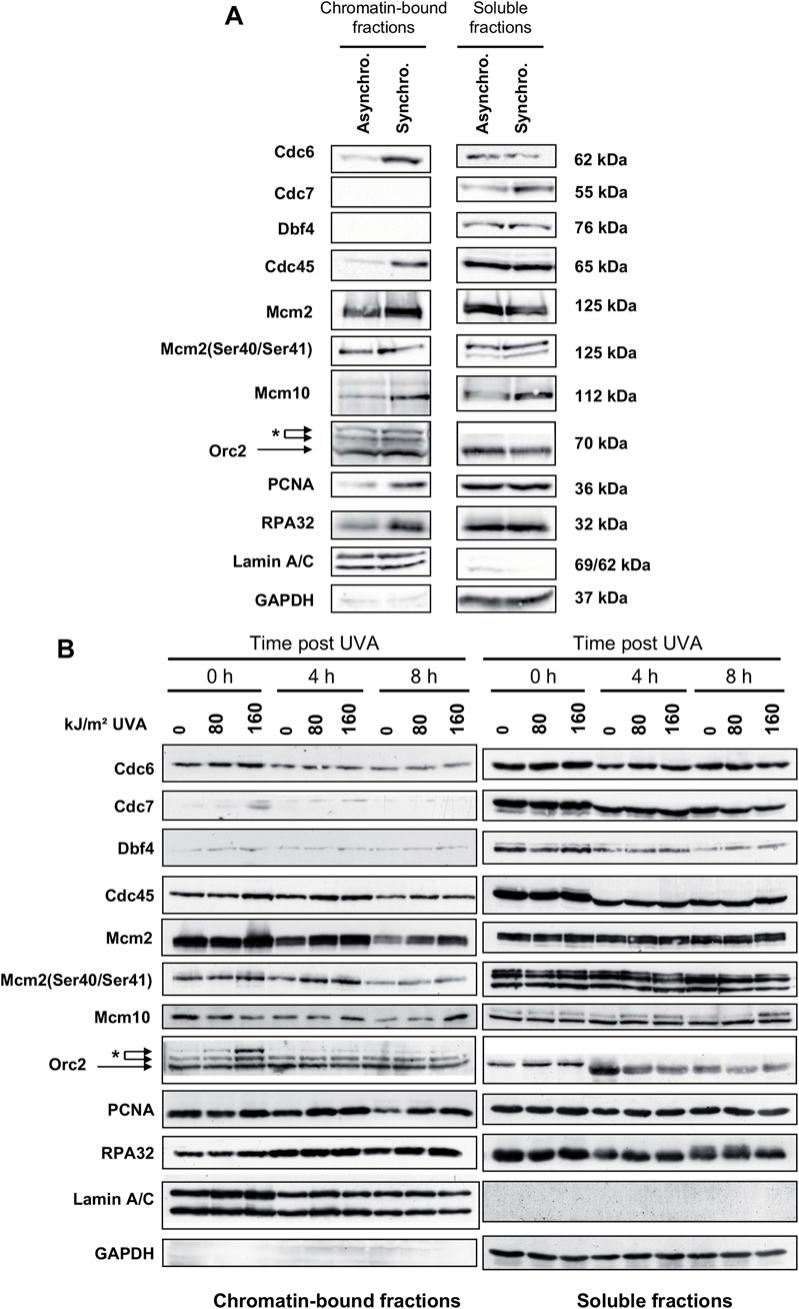
No relocalization or post-translational modifications of some components of the replication machinery after UVA radiation. Asynchronous MRC5Vi cells or synchronized in early S-phase (condition S0R, see Material and Methods) are untreated or exposed to 80 and 160 kJ/m^2^ of UVA radiation. (**A**) Subcellular localization of Cdc6, Cdc7, Dbf4, Cdc45, Mcm2, Mcm10, Orc2, PCNA, RPA32 in asynchronous cells (Asyncro.) or cells synchronized in early S-phase (Synchro.). (**B**) The fractions of chromatin-bound proteins and total soluble proteins were recovered at various time points post UVA. The expression levels of Cdc6, Cdc7, Dbf4, Cdc45, Mcm2, p-Mcm2(Ser40/41), Mcm10, Orc2, PCNA, and RPA32 were detected by Western blotting in denaturing and reducing conditions. Lamin A/C and GAPDH were used as loading control for the chromatin-bound and soluble fractions, respectively. The apparent molecular weight of each protein is indicated on the right side of each blot (panel 7A). The stars (*) indicate the position of the non specific bands detected by Orc2 antibody (H-300, SCBT).

As we observed a prolonged inhibition of the origin firing, we thought that UVA-induced ROS may destabilize or modify components of the DNA replication machinery. This process involves many players [60] that act in the formation of the pre-replication complex (pre-RC) at the end of mitosis, in its conversion into a pre-initiation complex (pre-IC) at the G1/S transition, and in the origin firing throughout S-phase[15,61]. As the sustained inhibition of DNA replication is observed when MRC5Vi cells are irradiated in S-phase but not when they are irradiated in G1 phase (see Fig 2B), we reasoned that components of the pre-IC might directly or indirectly be altered by UVA-induced ROS. Such alterations may result in a reduced binding to chromatin and/or to irreversible post-translational modifications of these proteins. Therefore, MRC5Vi cells were synchronized in early S-phase and exposed to increasing doses of UVA (condition S0R). Chromatin-bound protein fractions were prepared either immediately or at 4 and 8 h post radiation, and then analysed by Western Blot in reducing and denaturing conditions. At first, we looked at the recruitment at the chromatin of Cdc6, Cdc7, Dbf4, Cdc45, Mcm2, Mcm10, Orc2, PCNA and RPA32 in cells synchronized in early S-phase. We observed more Cdc6, Cdc45, Mcm2, Mcm10, PCNA and RPA32 proteins at the chromatin in early S-phase synchronous cells than in asynchronous cells, while Cdc7 and Dbf4 were not detected (Fig 7A). Note that we observed in the chromatin-bound fraction, but not in the soluble fraction, two extra bands that were detected by the anti-Orc2 antibody (H-300, SCBT) above the Orc2 band (Fig 7A). However, these two bands were not detected by other anti-Orc2 antibodies (shown in S6 Fig, see also [62,63]). We concluded that they correspond to non-specific cross reactivity of the H-300 antibody rather than to endogenous Orc2 modifications. In response to UVA radiation, none of the analysed proteins showed a reduced binding to chromatin in comparison to non-irradiated cells (Fig 7B). In marked contrast, 8 h post treatment, we observed more Cdc45, Mcm2, Mcm10, PCNA and RPA32 at the chromatin in UVA-treated cells than in non irradiated cells (Fig 7B). This is in agreement with the fact that at this time point, there are more cells in S-phase in UVA-treated cells than in non-irradiated cells. Surprisingly, the strongest changes were observed regarding the two non specific bands detected by the anti-Orc2 antibody (H-300, SCBT), whose intensities increased immediately after UVA radiation in a dose dependent manner (Fig 7B). Overall, the reduced origin firing after photosensitization by UVA cannot be simply explained by major modifications/relocalization of Cdc6, Cdc45, MCM2, Orc2, PCNA and RPA32.

## Discussion

Most UVA-mediated damage occurs indirectly through the absorption of UVA radiation by endogenous or exogenous non-DNA sensitizers, generating reactive oxygen species (ROS), especially singlet oxygen (^1^O_2_) [3–5,64]. UVA-induced ROS can cause damage to DNA, proteins and lipids (for reviews see [7–10]) and the concomitant modifications of these biomolecules contribute to the complexity of the cellular response to UVA [8]. In this study, we have shown that singlet oxygen-mediated oxidation during UVA treatment alters the dynamic of genomic DNA replication.

We demonstrated that vitamins present in MEMi are most likely the main photosensitizers that generate ^1^O_2_ upon exposure to UVA radiation (Fig 1) and our results using sodium azide point to ^1^O_2_ as a major player in the cellular response to UVA-induced ROS (this study, see also [33,38,65]). Within the dose range of UVA radiation used in our studies (*i*.*e*. ≤ 160 kJ/m^2^), the cellular responses are strongly attenuated in cells irradiated in PBS, a buffer devoid of extracellular photosensitizers. Such differences between PBS and MEMi can be explained by an efflux of the vitamins when the cells are treated in PBS, thus leading to a strong decrease of the intracellular concentration of vitamins and thus of intracellular photosensitizers. In MEMi, endogenous photosensitization reactions can reversibly or irreversibly oxidize several cellular proteins [38,65], including component(s) of the DNA replication machinery (this study), thus contributing to the slowdown of DNA replication.

A second non-exclusive hypothesis is to take into account the local concentration of vitamins at the cell membrane when cells are treated in MEMi *versus* PBS. Interestingly, we have previously reported that adding a photosensitizer such as naproxen (NAP, a non-steroidal anti-inflammatory drug) to PBS during irradiation led to the slowdown of DNA replication [5]. More importantly, we concluded from this study that the extent of inhibition of DNA replication after UVA radiation correlates with the extracellular NAP concentration rather than the intracellular NAP concentration [5]. These observations raise the idea that UVA radiation, *via* the production of ^1^O_2_, induces membrane damage that activates a signaling pathway which is a DNA damage-independent S-phase checkpoint. In favour of this hypothesis, we previously demonstrated that the three main kinases that control the cell cycle in response to DNA damage (*i*.*e*. ATR, ATM and p38α) [66] are dispensable to trigger the slowdown of DNA replication [33]. Furthermore, we did not observe arrested DNA replication in response to UVA-induced ROS (Fig 2B), a feature revealed after UVB radiation (a genotoxic agent) by the lack of BrdU incorporation in S-phase cells [67]. Interestingly, sphingosine, a lipid-signaling molecule, was described as a modulator of human translesion DNA polymerase activity [68] while oxidized membrane components (*e*.*g*. 4-hydroxynonenal, ceramides) generated by UVA-induced ROS are signaling molecules involved in the regulation of the expression of several genes [69].

We showed that the replication of genomic DNA was slowed down immediately after photosensitization by UVA radiation and that this effect lasted several hours (Fig 2). We and others have shown that the ROS level is maximal immediately after UVA radiation (shown in S2 Fig, see also [49]) to return to its basal level one hour later, suggesting that the long-lasting interference with DNA replication is established at the moment of ROS production. Replication of the whole genome throughout S-phase requires the activation of DNA replication origins and bidirectional elongation from activated origins [70]. We showed that UVA-induced ^1^O_2_ rapidly generates a transient reduction of the velocity of replication forks, with a full recovery 5 hours after irradiation (Fig 3). Interestingly, BrdU incorporation falls by 46% and 70% at 80 and 160 kJm^2^ of UVA, respectively (Fig 2), while the forks velocity drops by 43% and 74% at these doses (Fig 3). Therefore, the reduced incorporation of BrdU in S-phase cells immediately after UVA radiation (time slot from 0 to 30 min) is fully consistent with the idea of replication fork slowing. However, at longer times post radiation (>4-5h), BrdU incorporation was still low while the replication fork velocity has returned to its basal level, showing that inactivation of origin firing also contributes to slow down DNA replication.

Many intracellular factors are required for proper DNA chain elongation. Among the potential targets of UVA-induced ROS, we investigated the intracellular pool of dNTPs, and concomitantly a key regulator of their production, the ribonucleotide reductase (RNR) [71], since a reduction of their levels can induce the slowdown of DNA replication [51,72,73]. We observed a transient and moderate reduction in the intracellular levels of dATP, dCTP and dTTP (Fig 4), and a transient oxidation of RRM1 subunit of RNR (Fig 5). It is well known that UVA-induced ROS is expected to partly oxidize dNTPs, especially dATP to 2-OH-dATP and dGTP to 8oxo-dGTP [74,75]. However, the dGTP pool was not modified (Fig 4) suggesting that the moderate drop of dATP, dCTP and dTTP is not due to their oxidation. Anyhow, the magnitude and persistence of these effects cannot fully explain the immediate drop of replication fork velocity and its slow recovery. We thus conclude that the slow recovery of the fork velocity after UVA radiation is due to a failure to properly incorporate the dNTPs at the sites of ongoing DNA replication rather than to a failure to maintain an appropriate concentration of dNTPs.

As mentioned above, we also found that the RRM1 subunit of RNR is oxidized after photosensitization by UVA radiation and is reduced in a few minutes following the end of radiation by intracellular reducing systems other than those involving Trx1 or Grx1 (Fig 5). RRM1 oxidation (RRM1^ox^) relies on the production of singlet oxygen and the presence of intracellular GSH, and is characterized by an increase of the apparent molecular weight of the RRM1 subunit due to the formation of one or more disulfide bridges. We have recently reported that XRCC3, a DNA repair protein, behaves the same way [38] This suggests that, in our experimental conditions, ^1^O_2_- and GSH-dependent formation of disulfide bridges is an important mechanism to oxidize sulfhydryls in some cellular proteins. We also showed that Trx1, Grx1, RRM2 or p53R2 subunits are not involved in the formation of RRM1^ox^. We also excluded the formation of mixed disulfides between RRM1 and GSH due to the very low molecular weight of GSH (307.32 g/mol) that cannot account for the gain of ≈ 20–30 kDa in the apparent molecular weight of RRM1^ox^. It should be noted that Stubbe and co-workers have observed an altered conformation of the larger subunit of *E*. *coli* and human RNR (α subunit with an apparent molecular weight of 87 and 90 kDa, respectively) which migrates as a 110 kDa protein by SDS PAGE [76,77]. They proposed that this altered conformation is most likely the result of a crosslink between a cysteine in the C-terminus of α and a cysteine residue of its active site [77]. Thus, further experiments are required to determine whether RRM1 oxidation is due to inter- or intra-molecular disulfide bridges. Moreover, as the activity of mouse RNR is inhibited *in vitro* by H_2_O_2_ in a concentration-dependent manner [30], it is of interest to determine if, in our experimental conditions, oxidized RNR has an impaired activity.

We also observed long-lasting inhibition of origin firing in response to photosensitization by UVA radiation (Fig 6) that is independent of Chk1 expression (Table 1) despite its activation [33]. We suggest that UVA-induced ROS inhibit directly (by direct oxidation) or indirectly (*via* activation of a ROS-dependent signalling pathway) a firing factor that is loaded onto licensed origins during S-phase to trigger DNA synthesis. The firing of origins requires i) the loading onto licensed origins of several factors that are sequentially loaded in G1 and/or during S-phase (for reviews [17,18,78]) and ii) the activity of two kinases, S-phase specific cyclin-dependent kinase (S-Cdk) and Dbf4 dependent kinase Cdc7 (DDK), that are crucial to promote complex assembly and to coordinate the timing of replication initiation (for reviews [18,79,80]). We looked at the chromatin-bound fraction at different times after irradiation of some of these firing factors without, however, noting obvious variations (Fig 7). Nonetheless, we cannot exclude that more subtle modifications, like oxidations induced by singlet oxygen at Tyrosine, Tryptophane, Methionine and Histidine residues [81] or phosphorylation/dephosphorylation events, contribute to alter protein activity. Thus, a more exhaustive and detailed analysis of the proteins found at the replication origins and forks is required to unravel the mechanism of inhibition of origin firing by UVA-induced ROS.

In conclusion, we provide pieces of a complex puzzle that links the production of singlet oxygen to a defect in DNA replication. This defect can be explained by the direct oxidation and inactivation of DNA replication proteins and/or the activation of a non DNA damage S-phase checkpoint. In fact, the adverse effects of UVA radiation on genomic DNA replication will strongly depend on the radiation dose, on the presence or not of a photosensitizer in the course of irradiation, and on its subcellular localization. With respect to human health, it is important to note that photosensitization is not restricted to UVA radiation but also occurs with visible light [82] and a large number of clinical drugs are actually listed as photosensitizers [83]. UVA-induced DNA replication lengthening was observed in all the cell lines analysed so far, including human primary fibroblasts, mouse embryonic fibroblasts, and cancer cell lines (melanoma, colon cancer, breast cancer and hepatocellular carcinoma) (P.-M. Girard, unpublished data). Because in eukaryotes a perturbation of DNA replication is associated with genomic instability and cancer predisposition [84], it is particularly relevant to investigate whether this mechanism of DNA replication impairment that involves oxidation processes may contribute to UVA-induced photocarcinogenesis in human primary skin cells.

## Supporting Information

S1 Fig NaN_3_ prevents the slowdown of DNA replication induced by photosensitization upon UVA radiation in S-phase synchronized cells. (**A**) MRC5Vi cells were synchronized in early S-phase by aphidicolin and exposed in aphidicolin-free MEMi either immediately (condition S0R) or at 4 h after release from the aphidicolin block (condition S4R). The cells were then incubated in fresh complete medium for 8 h (condition S0R) or 4 h (condition S4R), trypsinized and fixed in 70% cold EtOH. (**B**) MRC5Vi cells in mid S-phase (condition S4R) were exposed to UVA radiation in PBS or in MEMi containing or not 10 mM NAC or NaN_3_. Following a further incubation for 4 h in NAC- and NaN_3_-free fresh complete medium, the cells were trypsinized and fixed in 70% cold EtOH. The cells were analysed by flow cytometry as a function of their DNA content (PI).
(EPS)

S2 Fig The production of ROS is maximal immediately after UVA radiation. MRC5Vi cells synchronized in S-phase were exposed or not to UVA radiation in MEMi. At various time points post radiation, complete medium containing 10 μM of the ROS probe DHR123 was added to the cells for 30 min. Thereafter, the cells were washed in PBS and fluorescence of the probe was monitored by flow cytometry.
(EPS)

S3 Fig NaN_3_ prevents the decrease of the replication forks velocity induced by UVA radiation. (**A**) MRC5Vi synchronized in early S-phase by aphidicolin were exposed or not to UVA radiation in mid S-phase (condition S4R). IdU and CldU labelings were performed at various time points (*i*.*e*. 0 h, ½ h, 1 h, and 4 h) after UVA radiation. Asynchronous (**B**) and synchronous (**C**) population of cells were untreated or exposed to 80 and 160 kJ/m^2^ of UVA radiation in the presence or absence of 10 mM NaN_3_. IdU and CldU labeling was performed sequentially immediately after radiation. To determine the impact of UVA radiation on fork velocity, only the CldU tracks length was scored. The values correspond to the mean of the forks velocity and were obtained from one experiment. A total of 900 to 1700 forks were analysed for these experiments. **P<0.001; *P<0.05 (two-tailed test).
(EPS)

S4 Fig Effect of 80 kJ/m^2^ of UVA radiation on the level of each intracellular dNTP. MRC5Vi synchronized in early S-phase by aphidicolin were exposed or not to 80 kJ/m^2^ of UVA in mid S-phase (condition S4R). The relative level of each dNTP was measured at the indicated time points post UVA radiation. The values represent the mean +/- sd of 5 independent experiments.
(EPS)

S5 Fig Inhibition of Chk1 expression does not prevent the slowdown of DNA replication induced by photosensitization through UVA radiation. (**A**) MRC5Vi cells were transiently transfected with control siRNA (siCtr) or Chk1 siRNA (siChk1) and 48 h post transfection; the cells were labeled with 10 mM BrdU for 30 min (T½h). Following an irradiation at 80 and 160kJ/m^2^ of UVA in MEMi, the cells were allowed to recover for 7.5 h in fresh complete medium (T8h), trypsined and fixed in 70% cold EtOH. The cells were analysed by bi-variable flow cytometry for BrdU incorporation (FITC anti-BrdU) and DNA content (propidium iodide, PI). (**B**) Western blot (WB) analysis of siCtr- and siChk1 transfected cells. Chk1 expression was detected using anti-Chk1 antibody. Actin was used as a loading control. The FACS and WB data are representative of two independent experiments.
(EPS)

S6 Fig Orc2 detection in the chromatin-bound and soluble fractions using 3 different antibodies. MRC5Vi cells were synchronized in early S-phase (condition S0R) and incubated in PBS (-) containing amino acid 1X (AA), vitamins 1X (Vit), 0.1 mg/ml riboflavin (Ribo), or in MEMi containing or not 10 mM NaN_3_. The cells were exposed to 160 kJ/m^2^ of UVA (+ UVA) or not (Ctr) and immediately lysed in lysis buffer. The * indicate the non-specific bands detected by Orc2 antibody from SCBT. Antibody H-300 maps to a region between residues 278 and 577. Antibodies A302-734 and A302-735 map to a region between residues 1 and 50, and 150 and 200, respectively.
(EPS)
